# Late antenatal care initiation: the case of public health centers in Ethiopia

**DOI:** 10.1186/s13104-018-3653-6

**Published:** 2018-08-06

**Authors:** Solomon Weldemariam, Ashenafi Damte, Kedir Endris, Melba C. Palcon, Kidisti Tesfay, Almaz Berhe, Tsige Araya, Hadgay Hagos, Haftom Gebrehiwot

**Affiliations:** 1 Department of Midwifery, College of Health Sciences, Mekelle University, Mekelle, Ethiopia; 2 Department of Nursing, College of Health Sciences, Mekelle University, Mekelle, Ethiopia

**Keywords:** Late antenatal care initiation, Public health centers, Tselemte district, Ethiopia

## Abstract

**Objective:**

The aim of this study was to determine the magnitude of late initiation of antenatal care visit and associated factors among antenatal care follow up women in Tselemte district health facilities. The data were obtained at health facilities level in a single survey within 1 month and there is no continuation part of this study or previously published part elsewhere.

**Results:**

60.5% of women were late to initiate the first antenatal care visit. Time constraint with household activity (24.4%), distance to health center (17.2%) and fear of long waiting time in health facility (19.5%) were among the reasons mentioned for late initiation of antenatal care visit. Monthly income ≤ $21(400 ETB) (AOR = 4.54, 95% CI 1.07, 19.33), women who accompanied by their husband during antenatal care visit (AOR = 6.99, 95% CI 2.82, 17.31), who had information access on antenatal care (AOR = 4.85, 95% CI 1.88, 12.50) and distance from home to health center (AOR = 5.44, 95% CI 1.54, 19.25) were significantly associated factors with late initiation of antenatal care visit. This study illustrated that large number of pregnant women still late for first antenatal care visit. Husband involvement and health education about the timing of antenatal care initiation should be encouraged in all aspects of maternal care.

**Electronic supplementary material:**

The online version of this article (10.1186/s13104-018-3653-6) contains supplementary material, which is available to authorized users.

## Introduction

Antenatal care (ANC) is a special care given for women during pregnancy and it is regarded as the cornerstone for improving peri-natal outcomes [1, 2]. The World Health Organization (WHO) focused antenatal care (FANC) model recommends a minimum of four ANC visit and the first visit to be within the first 16 weeks of gestation [3, 4]. Several studies illustrated that early and frequent ANC follow up provides an opportunity for institutional delivery [5, 6]. A pregnant women with untreated syphilis, 70–100% of their babies would be acquired this infection congenitally and one-third would end up with stillbirths [3, 4, 7]. It has been estimated that 25% of maternal deaths and 15% of life threatening complications occur during the prenatal period [7].

In developing countries, majority of pregnant women start the first ANC visit by their 2nd or 3rd trimester of pregnancy [8]. Hypertension and hemorrhage during pregnancy accounts’ for one-third to half of maternal deaths [7]. Demographic Health Servey findings showed that the variation in timing of ANC booking across sub-Saharan African remains notable. For example, only 11% of women in Ethiopia, 16% in Nigeria, 55% in Ghana, 12% in Kenya and 15% in Malawi have started ANC follow up by their first 12 weeks [9]. Several studies in Ethiopia report that late entry to ANC visit associated with socio-demographic variables, parity, media exposure, lack of social support, and cultural factors even though these studies are not consistent [3, 4, 10, 11]. The Ethiopian government has built an impressive work to improve the health of mothers through health extention workers [7, 12, 13]. There is also an improvement in ANC booking time from 6% in 2005 to 11% in 2011 [14]. Despite of these improvements, still maternal mortality ratio (MMR) is unacceptable in Ethiopia (676/100,000 births) [14]. As far as our knowledge, studies related to timing of ANC visit are scarce in the study region.

## Main text

### Methods

#### Study setting

Institution based cross-sectional study was conducted in Tselemti district, North-West Tigray Ethiopia, from Feburary 5 to March 3/2014. It is located to the Northern part of Ethiopia, about 1158 km from Addis Ababa and 375 from Mekelle the capital city of Tigray Region. According to 2007 central Statistics, Tigray region has an estimated total population of 4,316,988, of which 2,190,523 were female [15].

### Sample size and sampling techniques

A single population proportion formula (n = (Z α/2)2 P (1 − P)/d2) was used to estimate the required sample size. The following assumption were made while calculating the sample size. Population proportion of women who start ANC visit late after 16 weeks of gestation assumed to be 67.3% [16], 95% confidence interval, 5% (d = 0.05) margin of error, and expected non-response rate 10%. Overall, we recruited a sample size of 372 respondents. There were seven public health centers in this district and all of them were included in this study. Proportional to population size (PPS) allocation technique was used to allocate sample size to each health center. Systematic sampling technique was used to recruit eligible respondents. The value of “kth” was 2 for the health centers and 3 for the primary hospital. Registration of the clients at ANC was used as sampling fram and lottery method was used to select the first participant.

### Inclusion and exclusion criteria

Being pregnant mother and reside in the study area at least for 6 month were used as eligibility criteria to include in this study. Women who have unknown gestational age were excluded from this study.

### Data collection tool and procedures

A semi structured questionnaire was adapted in English from published literatures and translated into Tigrigna language (local language). A Pretest was conducted among 10% (38 women) of the sample size in Dabaguna Health Center outside of the study area which has similar socio-demographic characteristics as the study district. Four female diploma midwives involved in data collection after having 2 days training and data were collected through face to face interview. The overall supervision was carried out by the principal investigator and 2 supervisors daily. Ethical approval was obtained from Institutional Ethical Review Board (IERB) of Mekelle University, College of Health Sciences. A letter of permission was obtained from Tigray Regional Health Bureau to Tselemti district Health Bureau then to respective Health Facilities. Informed written consent was taken from each study participants and for participants below 18 years old was taken from their mothers/fathers.

### Statistical analysis

Data were cleaned and entered into SPSS version 20.0 software for processing and analysis. Descriptive statistics such as percent, mean and standard deviation were computed. Bivariate and multivariable logistic regression analysis were computed. Odds ratio with 95% CI was computed to ascertain the association between covariate and dependant variables. Variables that have P-value of < 0.2 at bivariate analysis were exported into multivariable logistic regression analysis to control possible confounding factors. Statistical test at P-value of < 0.05 were considered as cut off point to determine statistical significance.

## Results

### Socio-demographic characteristics

Out of 372 recruited respondents, 365 of them were responded completely, giving a response rate of 98.1%. The mean age of the respondents was 24.8 years (SD ± 5.8). Almost, 352 (96.4%) of respondents were Tigray in ethnicity and 350 (95.9%) Orthodox Christian. Regarding occupation, 251 (68.8%) husbands were farmer, 43 (11.8%) civil servant, 36 (9.9%) merchant and the rest were daily laborer by occupation (Table 1).

**Table 1:** Socio-demographic characteristics among pregnant mothers attending ANC service in Tselemte district, Tigray, Ethiopia, March 2014

Variables	Frequency (n = 365)	Percent (%)
Age (years)		
15–19	73	20
20–24	108	29.6
25–29	89	24.4
30–34	65	17.8
35–39	30	8.2
Marital status of the mother		
Married	351	96.2
Divorced	4	1.1
Separated	3	0.8
Cohabitated	7	1.9
Residence		
Urban	120	32.9
Rural	245	67.1
Educational status		
No formal education	214	58.6
Elementary (1–8)	99	27.1
Secondary (9–12)	34	9.3
Higher education	18	4.9
Occupational status		
House wife	100	27.4
Farmer	202	55.3
Civil servant	20	5.5
Business women	28	7.7
Daily laborer	15	4.1
House hold monthly income(ETB)		
≤ 400	94	25.8
401–600	81	22.2
601–1000	102	27.9
> 1000	88	24.1
Husband education		
No formal education	159	43.6
Elementary	143	39.2
Secondary	30	8.2
Higher education	33	9

### Reproductive and perception of ANC service characteristics

Out of 365 respondents, 269 (73.7%) were multi-gravida and 96 (26.3%) were primi-gravida. Almost all respondents, 357 (97.8%) were perceived that antenatal care check-up is essential to the health of mother and the fetus. One hundred fifty-two (41.6%) and 78 (21.4%) respondents perceived that 4 and > 4 ANC visits are mandatory during pregnancy respectively. Majority, 285 (78.1%) of respondents were inform their pregnancy to their husbands and the rest to their relatives and friends initially (Table 2). Majority of respondents (92.9%) reach the health center on foot and 242 (66.3%) had not radio or Television at home.

**Table 2:** Reproductive and perception of respondents characteristics among pregnant mothers attending ANC service in Tselemte district, Tigray, Ethiopia, March 2014

Variables	Frequency (n = 365)	Percent (%)
Parity (N = 365)		
Nulliparous	96	26.3
≥ 1	269	73.7
History of abortion (N = 365)		
No	334	91.5
Yes	31	8.5
History of still birth (N = 365)		
No	339	92.9
Yes	26	7.1
Means of diagnosing pregnancy (N = 365)		
Missed period	271	74.2
By urine test	94	25.8
Who planned the Px (N = 365)		
Wife	1	0.3
Husband	12	3.3
Both	339	92.9
Neither	13	3.6
Received advice when to start ANC for current P_X_ (N = 365)		
No	149	40.8
Yes	216	59.2
Source of Information for ANC (N = 365)		
Women development army	66	30.6
Health extension workers	64	29.6
Husbands and	51	23.6
Other family members	35	16.3
Decision maker (N = 365)		
Joint decision with spouse	179	49
Themselves	104	28.5
Husband	53	14.5
Their relatives	29	8

### Past history of ANC service utilization

Among the multi-gravida respondents, 192 (71.4%) of them had at least one history of ANC visit, of which 46.4% within the recommended time. About, 135 (70.3%) of respondents were receive information when to start ANC visit from health service providers and 17 (6.3%) of them had history of pregnancy complications. One hundred twelve (58.3%) of respondents had waiting time below 1 h, 53 (27.6%) between 1 and 2 h and the rest > 2 h in first visit.

### Magnitude of late ANC initiation and reason for late booking

Among the total study respondents, 221 (60.5%) of them were start their ANC follow up late (after 16 weeks of gestation). One-fourth (25.5%) of respondents initiated their ANC visit in first trimester, 67.8% in second trimester, and 6.9% in third trimester. Time constraint with household activity (24.4%), distance to health center (17.2%), perceived they were save (19.5%), unawared of pregnancy (7.2%), not perceived benefit (10%), Perceived the right time (5%), negligence (5.9%), getting service from HEWs (8%), fear of long waiting time (1.4%) and unwanted pregnancy (1.4%) were the reasons mentioned for late booking.

### Factors associated with late initiation of first ANC visit

The odds of late ANC initiation was 4.5 among ANC visitors who had monthly house hold income of ≤ $ 21(400 ETB) when compared to women with monthly house hold income of > $ 52(1000 ETB) (AOR = 4.54, 95% CI 1.07, 19.33). The odds of late ANC initiation was 6.99 among ANC visitors yet not accompanied by their husband to health center as compared to their counter parts (AOR = 6.99, 95% CI 2.82, 17.31). Mothers who lack information when to start ANC follow up was 4.85 more likely to start ANC follow up late compared to their counter parts (AOR = 4.85, 95% CI 1.88, 12.50). The likelihood of late ANC initiation was 5.44 among ANC attendees who walk more than 2 h to arrive health center than those who arrive at less than an hour walk (AOR = 5.44, 95% CI 1.54, 19.25) (Table 3).

**Table 3:** Factors associated with late first ANC visit among pregnant mothers attending ANC service in Tselemte Woreda, Tigray, Ethiopia, March 2014

Variables	Delay ANC visit		Crude OR (CI 95%)	Adjusted OR (CI 95%)
	No n (%)	Yes n (%)		
Educational status N = 365				
Illiterate	67 (31.3)	147 (68.7)	2.28 (1.48, 3.51)	2.39 (0.79, 7.21)
Literate	77 (51.0)	74 (49.0)	1	1
Occupational status N = 365				
House wife	44 (44)	56 (56)	1.94 (0.96, 3.91)	0.43 (0.10, 2.18)
Farmer	58 (28.7)	144 (71.3)	3.79 (1.97, 7.29)	0.23 (0.03, 1.54)
Civil servant and merchant	29 (60.4)	19 (39.6)	1	1
House hold monthly income in ETB N = 365				
≤ 400	27 (28.7)	67 (71.3)	3.12 (1.69, 5.76)	4.54 (1.07, 19.33)*
401–600	27 (33.3)	54 (66.7)	2.51 (1.35, 4.69)	3.40 (0.77, 15.12)
601–1000	41 (40.2)	61 (59.8)	1.87 (1.05, 3.33)	1.47 (0.38, 5.67)
> 1000	49 (55.7)	39 (44.3)	1	1
Parity N = 365				
Nulliparous	47 (48)	51 (52)	1	1
≥ 1	97 (36.3)	170 (63.7)	1.62 (1.01, 2.58)	0.52 (0.05, 5.92)
Number of child alive N = 365				
No child	57 (49.6)	58 (50.4)	1	1
01-Feb	45 (34.1)	87 (65.9)	1.90 (1.14, 3.17)	6.56 (0.56, 76.29)
≥ 3	42 (35.6)	76 (64.4)	1.78 (1.05, 3.01)	0.95 (0.09, 10.52)
Husband accompany to HC N = 365				
No	56 (27.2)	150 (72.8)	3.32 (2.14, 5.15)	6.99 (2.82, 17.31)*
Yes	88 (55.3)	71 (44.7)	1	1
Received advice on time of booking for index P_X_ N = 216				
No	31 (25.6)	90 (74.4)	3.51 (1.98, 6.24)	4.85 (1.88, 12.50)*
Yes	52 (54.7)	43 (45.3)	1	1
Time taken to health center (min) N = 339				
< 60	79 (62.2)	48 (37.8)	1	1
60–120	20 (39.2)	31 (60.8)	2.55 (1.31, 4.97)	1.12 (0.25, 5.05)
> 120	30 (18.6)	131 (81.4)	7.19 (4.21, 12.27)	5.44 (1.54, 19.25)*

* Significantly associated at P < 0.05

## Discussion

Despite of WHO recommendation that the first ANC visit to be within the first 16 weeks of gestation, high proportion of women were late. The current estimate is higher when compared to previous report from Addis Ababa, the capital city of Ethiopia, where 37.3% of women were start their first ANC visit late [17]. This might be due to the socio-demographic differences between these two regions. Health service accessibility, media exposure and educational status of the community might be in good position in Addis Ababa compared to the current study setting. However, the current finding is slightly lowered compared to finding from Mekelle (Ethiopia), where 67.3% of women were start their first ANC visit late [16]. The observed difference might be due to a relative good community sensitization and mobilization in utilizing maternal health service than ever by the health sector.

Women who have monthly income of less than 21 dollar more likely to present themselves late for first ANC visit. In Ethiopia, maternal health service are given free of charge however women may need money for food, transportation and for some laboratory investigation and drugs that are not available in public health centers. Even some women might not awarded the availability of free ANC service. Similar findings has been reported from Holeta (Ethiopia), and Ghana whereby mothers with house hold income of less than 23 USD were more likely to present late compared to women with house hold income of above 57 USD [18, 19].

The present study demonstrated that women who did not accompany by their husband during ANC follow up were more likely to present late for first visit compared to their counter parts. This is possibly due to the facts that in Ethiopia the husband is usually more influential, economically empowered and socially accepted than women. The late booking therefore is likely to be attributed to the lack of financial support or lack of encouragement from partners which could discourage women from seeking early ANC services. Similar findings from Mekelle and Hadya (Ethiopia) and Tanzania support the current finding [5, 8, 20]. Women who were not informed about the timing of ANC initiation were more likely to present late when compared to their counterparts. This might be, information is vital for development of health seeking behaviour timely. This finding was in line with the finding reported from Addis Ababa and Mekelle (Ethiopia) [16, 17]. Women who walked more than 2 h on foot to access the health facility were more likely to initiate ANC visit late compared to those who walked less than an hour. Distance is a significant factor in many studies from Africa. For example, a study done in Zambia, Hadya and Yem (Ethiopia) [20–22] were consistent with the current finding. The possible explanation might be most respondents of this study were reside in mountainous areas with poor infrastructure, and lack of transportation with high costs for transportation.

## Conclusion and recommendation

The current finding illustrat that highest proportion of women in the study area still late for first ANC visit which in turn implies that many women are at risk of several obstetric complication. Socio-demographic variables still playing a great role in hindering maternal health service utilization.

Midwives, and other health cadres should strengthen the sensitization and mobilization of mothers on the importance of timely initiation of ANC services. Regional Health office also should open mobile clinics and home care visits program to reach those mothers disadvantaged due to geographical barrier. Husband involvement during ANC service follow up should be encouraged by midwives.

## Limitation

Since this study conducted in one district health facilities, the findings might not be generalizable to the entire community in the region. Recall bias might be there since gestational age was determined based on the women memory.

## Additional file

**Additional file 1.** An English version questionnaire used to measure this findings and it was developed from different published literatures and adjusted contextually.

## Abbreviations

ANC: antenatal care
WHO: World Health Organization
WDA: women development armey
HEWs: health extension workers
USD: USA dollar
