# Supplementary material for: Late antenatal care initiation: the case of public health centers in Ethiopia
Source: BMC Res Notes. 2018 Aug 6;11:562. doi: 10.1186/s13104-018-3653-6 (PMC6080357; doi:10.1186/s13104-018-3653-6)
Supplement: Supplementary file 1 — Additional file 1. An English version questionnaire used to measure this findings and it was developed from different published literatures and adjusted contextually. [file 13104_2018_3653_MOESM1_ESM.docx]

## English version questionnaire

Mekelle University, College of Health Sciences, Department of Midwifery Survey Questionnaire, to assess level of late initiation of first ANC and associated factors among mothers who attend ANC at health facility of Tselemte district, North-West, Tigray, Ethiopia, 2014.

**Part I: Socio demographic characteristics of respondents**

| **S. NO** | **Questions** | **Response** | **Skip pattern** |
| --- | --- | --- | --- |
| 101 | Age | In years--------------- |  |
| 102 | Marital status | 1. Married  2. Divorced  3. Widowed  4. Single  5. Separated | Skip Q 109 & 110 if the answer Q 102 is out of 1 married |
| 103 | Ethnicity | 1. Tigray  2. Amhara  8. Others, specify ________ |  |
| 104 | Residence | 1. Rural  2. Urban |  |
| 105 | Educational Status | 1.Unable to read and write  2. Able to read and write  3. Primary education(1-8)  4. Secondary education(9-12)  5. College or University |  |
| 106 | Occupation status | 1. House wife  2. Farmer  3. Civil servant  4. Business women  5. Daily Laborer  7. Others, specify __________ |  |
| 107 | Religion | 1. Orthodox  2. Muslim  3. Protestant  4. Catholic  5. Others(specify)______ ­­­­­­­ |  |
| 108 | House hold monthly income(Ethiopian birr) | ______________________(ETB) |  |
| 109 | Husband’s educational Status (If married) | 1.Unable to read and write  2. Able to read and write  3. Primary education(1-8)  4. Secondary and above (9-12)  5. College or University |  |
| 110 | Husbands occupation (If married) | 1. Farmer  2. Governmental employee  3. Private organization Employee  4. Merchant  5. Daily laborer  6. Other, specify___________ |  |
| 111 | Do you have any of the following means of communication? | 1. Radio  2. TV  3. None |  |
| 112 | Means of transportation? | 1. Public transport 2. On foot |  |
| 113 | Time taken to reach health facility from home? | _______________minutes |  |

**Part II: Reproductive characteristics and perception of respondents on ANC**

| 201 | | How many times you have pregnant including abortion | --------------- |  |
| --- | --- | --- | --- | --- |
| 202 | | How many births have you ever had births (births that occurred after 28 weeks) (parity)? | ----------------- |  |
| 203 | | Number of children alive? | ______________________ |  |
| 204 | | Did you experience abortion(termination of pregnancy <28 weeks | ----------------- |  |
| 205 | | Number of still births ever had(birth of dead fetus after 28 weeks of gestation) | ----------------- |  |
| 206 | | How did you diagnose your current pregnancy? | 1. Missed period(menses 2. Urine test at health facility |  |
| 207 | | Who planned the Px | 1.Wife  2. Husband  3. Both  4. Neither |  |
| 208 | | Did you receive information when to start ANC? | 1. YES 2. No |  |
| 209 | | From where you get information for ANC follow up? | 1. Women development army 2. Health extension workers 3. Health professions 4. Husbands 5. Other family members |  |
| 210 | | To whom did you inform your pregnancy initially? | 1. To health profession 2. To her husband 3. To her family 4. To relatives |  |
| 211 | | What is the advantage of ANC? | 1. For the health of fetus 2. For the health of mother 3. For both the health of mother and fetus |  |
| 212 | | How many visits of ANC pregnant women should have? | ----------------- |  |
| 213 | | Did your husband accompany you to health facility during ANC visit? | 1. Yes  2. No |  |
| 214 | | Who decide for maternal health service care utilization at home? | 1. Husband  2. wife  3. both  4. relatives/family |  |
| **Past history of ANC** | | | | |
| 301 | | Did you have history of ANC in the previous pregnancy? | **1. Yes**  **2. No** | **If no skip to Q 305** |
| 302 | | If yes, how many visits did you have? | **_______________________** |  |
| 303 | | At what month of your pregnancy did you start ANC visit? | **_____________________** |  |
| 304 | | During ANC follow up did you get information about when to start the first ANC visit? | 1. Yes  2. No |  |
| 305 | For how many minutes did you wait in health facility in first visit before you get the service providers? | | ____________________minutes |  |
| 306 | Did you have any complication in that pregnancy?(APH, Infection, preeclampsia & eclampsia, hyperemesis gravida rum etc..) | | 1. Yes  2. No |  |
| **Timing of the first ANC visit and reasons for delay for the current pregnancy** | | | | |
| 401 | When was your last normal menstrual period(LNMP) | | Date____/month___/year_______ |  |
| 402 | Date of the interview?(for second or more visit review their charts for date of first visit ) | | Date____/month___/year_______ |  |
| 403 | Calculate the gestational age at first visit? | | _____________weeks |  |
| 404 | Does the first visit after 16 weeks? (Based on above information) | | 1. Yes  2. No | **If no stop here** |
| 405 | If yes for the above question what were the reasons?(More than one reason possible) | | 1. Busy at house hold activity  2. due to long distance to health facility  3. I have no problem with my pregnancy  4. I did not aware that I was pregnant  5. I have knowledge about the advantage of early ANC  6. I consider this time the right time for first visit  7. Negligence  8. I have got the first service from health extension workers  9. fear of long waiting time in health facility  10. I were not sure whether to continue with this pregnancy because I did not need this pregnancy  11. others reasons, specify-------- |  |
